# Testosterone regulates the expression and functional activity of sphingosine‐1‐phosphate receptors in the rat corpus cavernosum

**DOI:** 10.1111/jcmm.13416

**Published:** 2017-12-20

**Authors:** Jing Yin, Yu‐ming Guo, Ping Chen, He Xiao, Xing‐huan Wang, Michael E DiSanto, Xin‐hua Zhang

**Affiliations:** ^1^ Department of Rehabilitation Zhongnan Hospital of Wuhan University Wuhan China; ^2^ Department of Urology Zhongnan Hospital of Wuhan University Wuhan China; ^3^ Surgery and Biomedical Sciences Cooper Medical School of Rowan University Camden NJ USA

**Keywords:** sphingosine‐1‐phosphate, testosterone, corpus cavernosum, smooth muscle, erectile dysfunction

## Abstract

The bioactive lipid sphingosine‐1‐phosphate (S1P) regulates smooth muscle (SM) contractility predominantly *via* three G protein‐coupled receptors. The S1P1 receptor is associated with nitric oxide (NO)‐mediated SM relaxation, while S1P2 & S1P3 receptors are linked to SM contraction *via* activation of the Rho‐kinase pathway. This study is to determine testosterone (T) modulating the expression and functional activity of S1P receptors in corpus cavernosum (CC). Adult male Sprague‐Dawley rats were randomly divided into three groups: sham‐operated controls, surgical castration and T supplemented group. Serum S1P levels were detected by high‐performance liquid chromatography. The expression of S1P1‐3 receptors and sphingosine kinases was detected by real‐time RT‐PCR. *In vitro* organ bath contractility and *in vivo* intracavernous pressure (ICP) measurement were also performed. T deprivation significantly decreased ICP rise. Meanwhile, surgical castration induced a significant increase in serum S1P level and the expression of S1P2‐3 receptors by twofold (*P *<* *0.05) but a decrease in the expression of S1P1 receptor. Castration also augmented exogenous phenylephrine (PE), S1P, S1P1,3 receptor agonist FTY720‐P contractility and S1P2‐specific antagonist JTE013 relaxation effect. T supplemented could restore the aforementioned changes. We provide novel data that castration increased serum S1P concentration and up‐regulated the expression of S1P2‐3 receptors in CC. Consistently, agonizing S1P receptors induced CCSM contraction and antagonizing mediated relaxation were augmented. This provides the first clear evidence that S1P system dysregulation may contribute to hypogonadism‐related erectile dysfunction (ED), and S1P receptors may be expected as a potential target for treating ED.

## Introduction

Penile erection is a neurovascular event involving the relaxation of corpus cavernosum smooth muscle (CCSM), which is maintained tonically contracted state in majority of the time 1, 2, 3. Although many studies have investigated CCSM relaxation pathways, few have focused on the role of the contractile apparatus in erectile function (EF). Similarly, the major therapeutic treatments available for ED have primarily targeted CCSM relaxation pathways with particular emphasis on NO/cyclic guanosine monophosphate (cGMP) signalling 3, 4, 5.

Shingosine‐1‐phosphate (S1P), a member of a large family of lipid metabolites termed sphingolipids, represents one of the key latest additions to the list of ‘vasoactive’ substances that modulate vascular tone 6. S1P is produced by sphingosine kinases (SphK) catalyzing the ATP‐dependent phosphorylation of sphingosine. Thus far, two different SphK isoforms have been found in mammals named SphK1 and SphK2 7. SphK1 is predominantly cytosolic and pro‐survival, while SphK2 functions mainly in the endoplasmic reticulum and stimulates apoptosis but its role is still poorly understood 6, 7, 8. In plasma, S1P can reach a concentration of 0.1–4 μM 9. Besides vasoactive potential, S1P is capable of regulating a wide array of biological processes such as cell proliferation, migration, survival, differentiation and others 6, 8, 10. Many of these cellular responses are initiated by S1P binding to and activating a set of five G protein‐coupled S1P receptors (S1P1–5) 11. In mammals, S1P1, S1P2 and S1P3 are found in all tissues, whereas S1P4 is restricted to lymphoid tissues and lung 12, and S1P5 to brain and skin 13. In blood vessels, vascular endothelial cells (ECs) and SM cells express specific receptors for S1P that modulates vascular tone 14. In particular, vasorelaxation is elicited by S1P through S1P1 receptors in ECs *via* endothelial nitric oxide synthase (eNOS) pathways 6, 8, 15, whereas S1P2 and S1P3 receptors in SM can elicit vasoconstriction responses through the activation of RhoA/ROK pathways 6, 8, 16, 17. For a given blood vessel preparation, whether S1P stimulation causes vasodilatation or vasoconstriction may depend on multiple experimental variables, such as the animal species, the vascular bed, the S1P concentrations, S1P receptor subtype expression profile, as well as disease states. In general, higher concentration of S1P induces vasoconstriction in resistance vessels such as mesenteric, cerebral and coronary arteries but has little or no effect on conduit vessels such as aorta, carotid and femoral arteries 6, 8. Apart from resistant arteries, S1P can contract SM of urinary bladder, uterus, gastrointestinal tract and bronchial tube 16, 18. With regard to CCSM, di Villa Bianca *et al*. 19 found exogenous S1P did not directly relax or contract of human CCSM strips but potentiated acetylcholine response. The tissues used in their study were obtained from male to female transsexual patients with antiandrogen and oestrogen pre‐surgery treatment. Thus, we do not know S1P on intact CCSM. In fact, we recently reported that S1P caused strong *in vitro* and *in vivo* contraction of CCSM of normal rats and patients with various kinds of ED, while S1P receptor antagonist induced CCSM relaxation 20.

Androgens play a dual role in the erectile process by controlling both pro‐erectile and anti‐erectile signalling pathways 21, 22. Indeed, testosterone (T) modulates NO and its generating enzyme NOS, which induce penile erection 23, 24, 25. Recently, our serial studies together with other institutions have confirmed that T also regulates phosphodiesterase‐5 (PDE5) 4, 5, 22, 26, 27, 28 which is mainly responsible for penile detumescence. In addition, castration up‐regulates CCSM contractility process, including hyper‐responsiveness to α‐adrenergic agonist, increased SM myosin (SMM) phosphorylation and alteration of SMM isoform composition, as well as activation of RhoA/ROK signalling 3, 29, 30.

However, the CCSM, unlike most other SM, spends the majority of its time in the contracted state and relaxes only upon receiving erectogenic stimuli. A better understanding of androgens that regulate contraction in the CCSM is as critical as their modulation on CCSM relaxation mechanism. The goal of this study was to investigate T modulating the expression and functional activity of S1P receptors in CCSM.

## Materials and methods

### Animals and tissues

Adult male Sprague–Dawley rats weighing 300–350 g were used in this study. All the rats were divided into three groups: sham, castration and castration with T (testosterone propionate, Sigma‐Aldrich; St. Louis, MO, USA). As described in previous study 31, rats that underwent just a perineal incision served as sham. Castration was performed through the bilateral orchiectomy under sodium pentobarbital anaesthesia. Part of the castrated rats was injected subcutaneously with 30 mg/kg T per week, and others were treated with vehicle (sesame oil) only. Rats were killed 2 weeks post‐surgery, blood was drawn from the heart for S1P measurements and CC was collected for organ bath physiology studies (placed in Krebs–Henseleit solution) or subsequent molecular analyses (frozen in liquid nitrogen). The prostate and seminal vesicles were harvested and weighted after kill. All animal studies were approved by the research committee of Zhongnan Hospital of Wuhan University.

### High‐performance liquid chromatography

S1P serum levels were determined with high‐performance liquid chromatography (HPLC) as previously described with minor modification 17. Briefly, serum samples were extracted into chloroform/methanol, and the obtained lipid samples were vacuum dried. The sphingolipids were then derivatized with 9‐fluorenylmethyl chloroformiate (FMOC‐Cl), and chromatographic detection of sphingolipids was performed with reversed‐phase chromatography on a 300 × 3.9 mm Delta‐Pak C18 column (Waters Corporation, Milford, MA, USA).

### Total RNA extraction and real‐time reverse transcriptase polymerase chain reaction (real‐time RT‐PCR)

As previously reported 17, 32, total RNA was isolated from the frozen tissues using TRIzol reagent (Invitrogen, Carlsbad, CA, USA) according to the manufacturer's protocol. RNA concentration and purity were determined using a ND‐1000 Nanodrop spectrophotometer (NanoDrop Technologies; Wilmington, DE, USA). For each sample, 1 μg of RNA was converted to complementary DNA (cDNA) using reverse transcriptase *via* the SuperScript II First‐Strand Synthesis System (Invitrogen) according to the manufacturer's protocol. Primer pairs were designed using published cDNA sequences obtained *via* Entrez Nucleotide of the National Center for Biotechnology Information (NCBI) and the Primer Express program (Applied Biosystems, Foster City, CA, USA). Amplicons were deliberately kept at between 50 and 100 base pairs (bp) for all primer pairs to enable equal transcriptional efficiency.

RT products then were amplified in a 96‐well plate in a 25 μl reaction volume with all samples run in triplicate, using the Model 7300 real‐time thermocycler (Applied Biosystems). The following experimental protocol was utilized: denaturation (95°C for 10 min. to activate the polymerase) followed by an amplification program repeated for 40 cycles (95°C for 15 sec., then 60°C for 60 sec.) using a single fluorescence measurement. The following targets were amplified using SYBR Green for amplicon detection: S1P receptors 1–3 and SphK1‐2. For relative quantification, the efficiency of amplification for each individual primer pair was determined using cDNA target and the 2^−ΔΔct^ method 33 in conjunction with the RQ Study Software version 1.2.3 (Applied Biosystems). Gene expression was normalized to expression of the RPL19 housekeeping gene.

### *In vitro* organ bath studies

As previously reported 31, 34, the rat CC was mounted longitudinally in a 4 ml organ bath—Multi Myograph Model 800MS (Danish Myo Technology, Aarhus, Denmark) by securing to the two pins. One of the pins was attached to a force transducer which was calibrated to mg of force prior to the start of experimentation. The myograph was connected in line to a PowerLab 4/30 data acquisition system (ADInstruments; Colorado Springs, CO, USA) and in turn to a dual‐core processor Pentium computer for real‐time monitoring of physiological force.

The SM strips were equilibrated for at least 1 hr in Krebs buffer maintained at a mean temperature of 37 ± 0.05°C with continuous bubbling of 95% O_2_ and 5% CO_2_ with buffer changes every 15 min. The buffer had the following mM composition: NaCl 110, KCl 4.8, CaCl_2_ 2.5, MgSO_4_ 1.2, KH_2_PO_4_ 1.2, NaHCO_3_ 25 and dextrose 11. The strips were continuously adjusted to a 400 mg resting tension and isometric tension recorded. After equilibration, the tissues were contracted with 60 mM KCl. This degree of contractile response was taken as 100% and the force induced by different concentrations of the various agonists including PE, S1P (Cayman Chemical, Ann Arbor, MI, USA) and FTY720‐P (Echelon Biosciences Inc, Salt Lake City, UT, USA), was expressed as a percentage of this value. Meanwhile, strips were pre‐contracted with 1 μM PE (a dose that was determined to induce remarkable contraction) and allowed to reach a stable tension, and then, the relaxant effects of increasing doses (1–10 μM) of S1P2 receptor‐selective antagonist JTE‐013 (Tocris, Ellisville, MO, USA) were evaluated.

### *In vivo* studies

As previously reported 4, 5, part of rats from each group was anaesthetized with pentobarbital (35 mg/kg) *via* an intraperitoneal (i.p) injection. Mean arterial pressure (MAP) *via* carotid artery and ICP were continuously monitored, using methods described previously 35. Briefly, an incision was made in the perineum, and a window was made in the ischiocavernosus muscle to expose both CC. The right crura were perforated with a 28‐gauge needle connected to PE‐50 tubing for ICP recording, while the left crura were perforated with a 30‐gauge needle connected to PE‐10 tubing for drug delivery. The cavernous nerve was identified ventrolateral to the prostate gland and carefully isolated. Direct electrostimulation (ES) of the cavernous nerve was performed with a delicate stainless steel bipolar hook electrode attached to the multijointed clamp. Each probe was 0.2 mm in diameter with a 1 mm separation between the two poles. Monophasic rectangular pulses were delivered by a signal generator (custom‐made and with built‐in constant current amplifier). MAP and ICP were recorded *via* pressure transducers connected in line to the PowerLab 4/30 data acquisition system connected in turn to computer for real‐time monitoring of pressure changes. Pressure transducers were calibrated to water prior to each experiment. After a stable baseline ICP was obtained, ICP rise induced by ES (width 5 ms, duration 30 sec., 2.5 V) at varying frequencies (1, 2, 4, 8, 16 Hz) was recorded. For another group of rats, intracavernous injection (ICI) of 50 μl ethanol was carried out to test vehicle effects. Then, increasing doses (50, 250, 500 nmols) of JTE‐013 alone were ICI with 30‐min. intervals between with washout (saline flush). The ICP rise was quantified by calculating the ratio of maximum ICP/MAP ×100.

## Results

**Figure 1:**
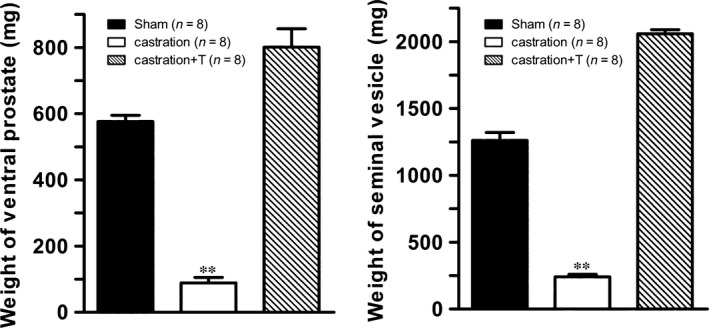
Effect of testosterone (T) on the weight of androgen‐sensitive organs. Left panel is bar graph of the average weight of the ventral prostates from all experimental groups. And right panel is bar graph of the average weight of seminal vesicle from all experimental groups. Values are expressed as means ± S.E. **P <* *0.01 *versus* sham or castration + T. (*n* = number of samples from different animals).

After a 2‐week surgical castration, obvious atrophy of androgen‐sensitive organs was observed in castrated rats. As shown in Figure 1, the weight of both prostate and seminal vesicles decreased by approximately eightfold and 10‐fold in castrated rats and the mass reinstated completely after T supplementation.

**Figure 2:**
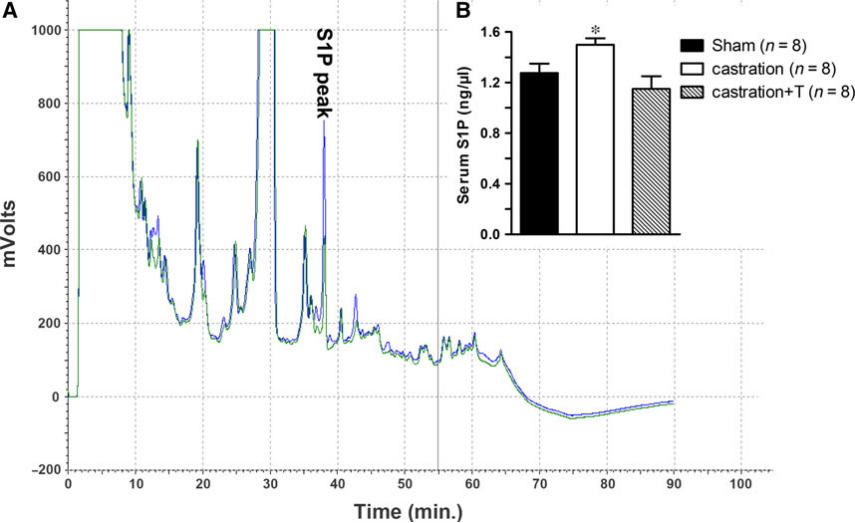
Effect of testosterone (T) on rat serum S1P levels. **A**: Serum S1P levels were detected by high‐performance liquid chromatography (HPLC). The green curve is serum sample, while the blue one is serum sample mixed with 2.6 ng exogenous S1P. **B**: bar graph of the average serum S1P from all experimental groups. Values are expressed as means ± S.E. *P <* *0.05 *versus* sham or castration + T. (*n* = number of samples from different animals).

Serum S1P concentrations were detected with HPLC. As displayed in Figure 2A, serum S1P peak appeared at 38 min. (green curve) which was confirmed by a synergic peak occurred exactly at the same time‐point when sample mixed with exogenous S1P (blue curve). As shown in Figure 2B, T deprivation significantly increased serum S1P to 1.5 ng/μl, which was 0.23 ng/μl higher than that of sham. And the change was reversed with T injection.

**Figure 3:**
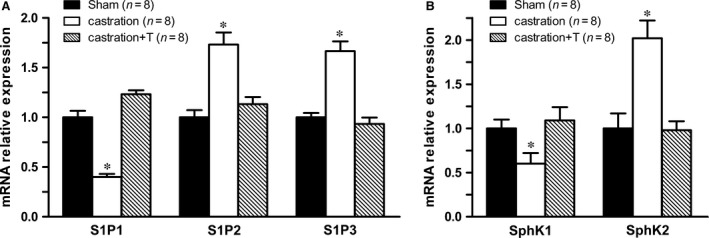
Expression of S1P receptors and sphingosine kinases (SphKs) in rat corpus cavernosum from all experimental groups. The expression of all studied molecules was quantified by real‐time RT‐PCR and normalized to expression of the ribosomal protein L19 (RPL19) housekeeping gene. **A**: S1P1‐3 receptors mRNA level. **B**: SphK1‐2 mRNA level. Values are expressed as means ± S.E. *P <* *0.05 *versus* sham or castration + T. (*n* = 8 different rats for each group).

To determine whether T affected the expression of S1P receptors in CC, the mRNA levels of S1P1‐3 receptors in CC were detected. With real‐time RT‐PCR, we found castration significantly increased both S1P2 and S1P3 transcripts in CC (Fig. 3A), which were mainly related to constriction of SM. In contrast, S1P1 mRNA was found to be significantly decreased in CC from castrated rats (Fig. 3A), which was contributed to vasorelaxation. Additionally, critical enzymes mediating the biosynthesis of S1P in CC were examined. As shown in Figure 3B, lower SphK1 and higher SphK2 mRNA levels in CC were found in castrated rats as compared with controls. Altered expression of all these molecules was maintained or recovered to control levels when T was given back.

**Figure 4:**
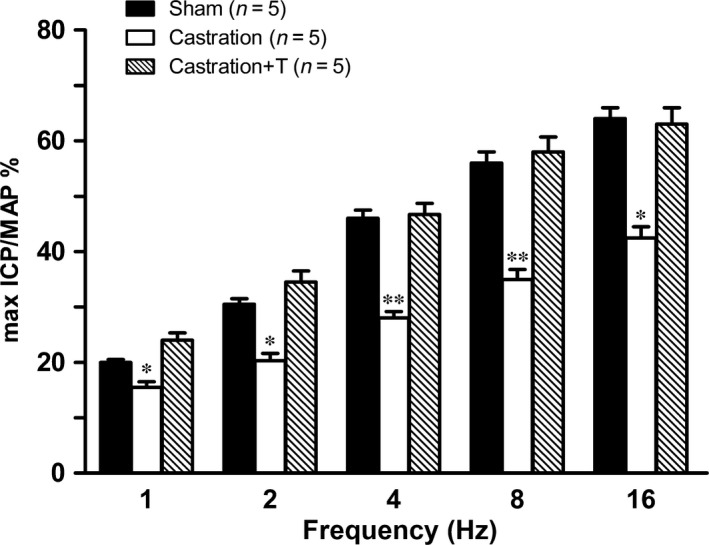
Effect of testosterone (T) on electrostimulation (ES)‐induced erectile response of rats. Penile erection was elicited by ES (width 5 ms, duration 30 sec., 2.5 V) of the rat cavernous nerve at varying stimulation frequencies (1–16 Hz). Erectile function was quantified by calculating maximal intracavernous pressure (ICP)/mean arterial pressure (MAP) ratio (×100). Values are expressed as means ± S.E. *P <* *0.05 *versus* sham or castration + T. **P <* *0.01 *versus* sham or castration + T. (*n* = 5 different rats for each group).

**Figure 5:**
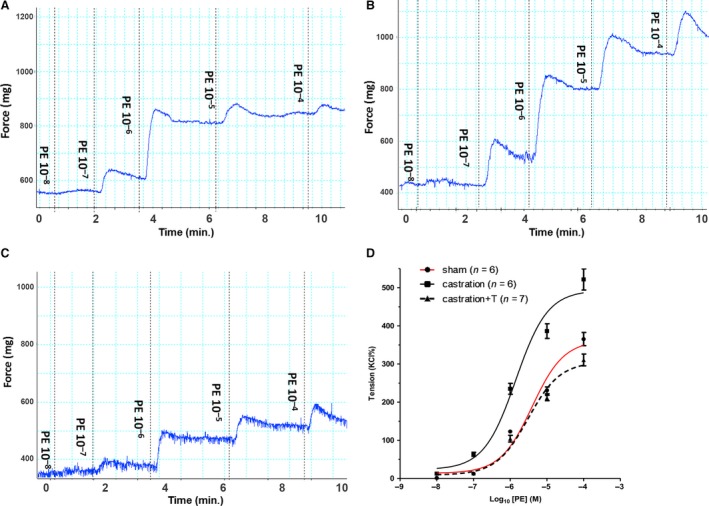
Phenylephrine (PE)‐induced contraction of rat corpus cavernosum smooth muscle (CCSM) *in vitro*. Panel **A**,** B** and **C** are PE‐induced dose–response force tracings of rats CCSM from sham (**A**), castration (**B**) and castration + T (**C**) groups, respectively. For typical tracings, the *x*‐axis represents time (min.), while the *y*‐axis represents force (mg). Panel **D** is summary graph for curves of PE‐induced dose–response contraction that resulted in sigmoid (S‐shaped) curves after logarithmic transformation. Maximal response to KCl was taken as 100%, while the contractility of PE was evaluated as a percentage of this response. Values are expressed as means ± S.E.. (*n* = CC strips obtained from 6 to 7 different rats for each group).

**Figure 6:**
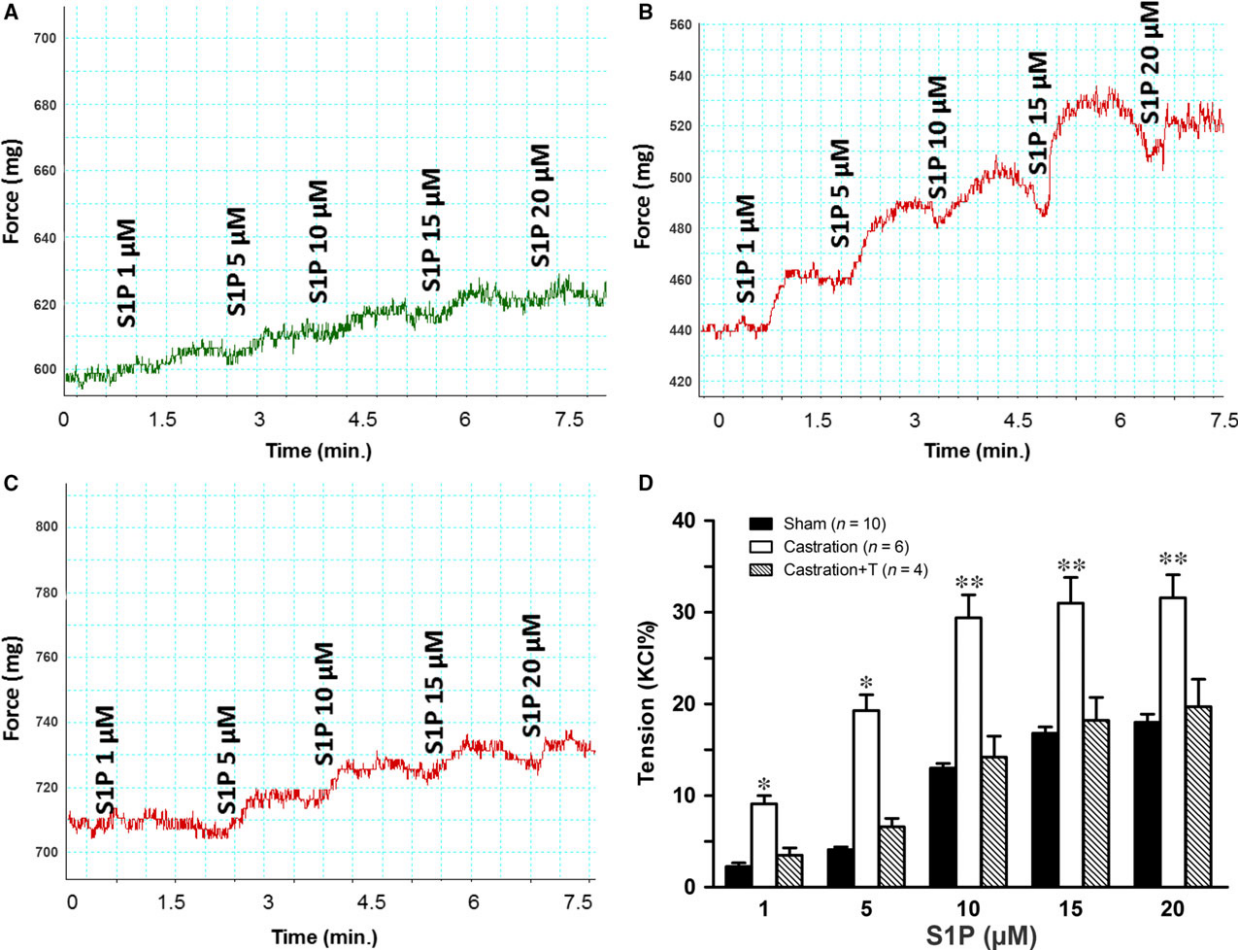
S1P‐induced contractility of rat corpus cavernosum smooth muscle (CCSM) *in vitro*. Panel **A**,** B** and **C** are S1P‐induced dose–response force tracings of rats CCSM from sham (**A**), castration (**B**) and castration + T (**C**) groups, respectively. For typical tracings, the *x*‐axis represents time (min.), while the *y*‐axis represents force (mg). Panel **D** is a summary graph for the data shown in panel **A–C**. Maximal response to KCl was taken as 100%, while the contractility of S1P was evaluated as a percentage of this response. Values are expressed as means ± S.E. *P <* *0.05 *versus* sham or castration + T. **P <* *0.01 *versus* sham or castration + T. (*n* = CC strips obtained from 4 to 10 different rats for each group).

**Figure 7:**
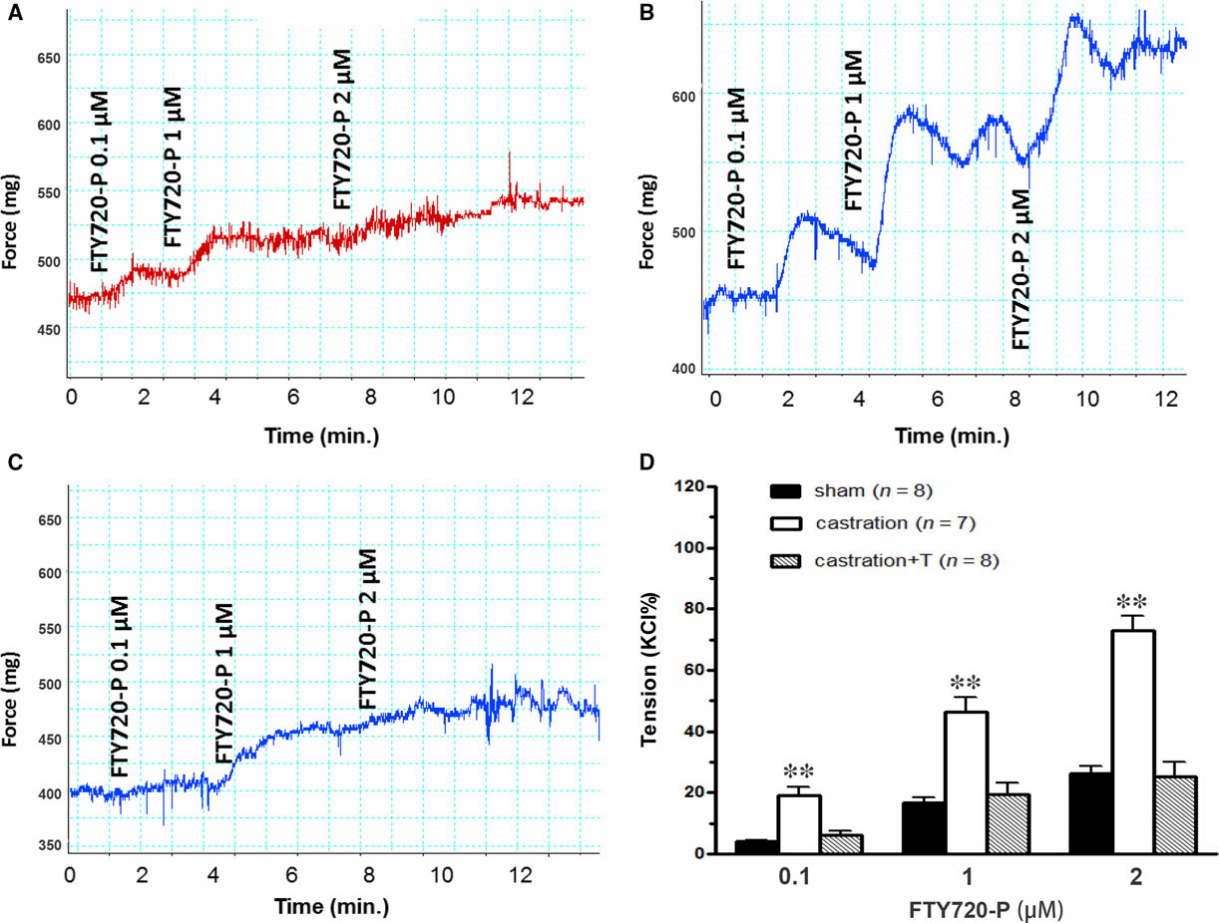
S1P analogue (FTY720‐P)‐induced contractility of rat corpus cavernosum smooth muscle (CCSM) *in vitro*. Panel **A**,** B** and **C** are FTY720‐P‐induced dose–response force tracings of rats CCSM from sham (**A**), castration (**B**) and castration + T (**C**) groups, respectively. For typical tracings, the *x*‐axis represents time (min.), while the *y*‐axis represents force (mg). Panel **D** is a summary graph for the data shown in panel **A**–**C**. Maximal response to KCl was taken as 100%, while the contractility of FTY720‐P was evaluated as a percentage of this response. Values are expressed as means ± S.E. **P <* *0.01 *versus* sham or castration + T. (*n* = CC strips obtained from 7 to 8 different rats for each group).

**Figure 8:**
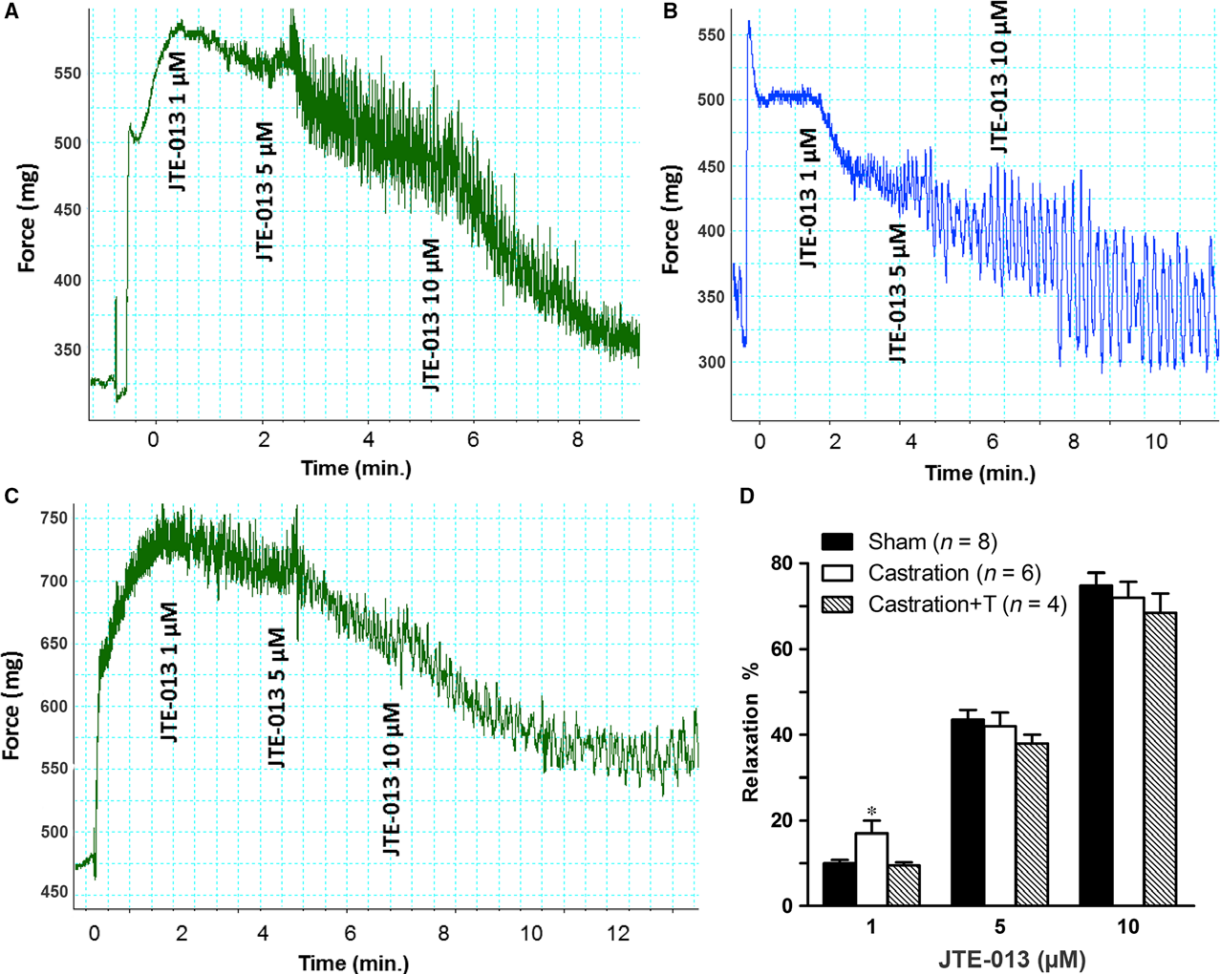
S1P receptor antagonists (JTE‐013)‐induced relaxation of rat corpus cavernosum smooth muscle (CCSM) *in vitro*. Panel **A**,** B** and **C** are JTE‐013‐induced dose–response relaxation force tracings of rats CCSM pre‐contracted with 1 μM phenylephrine (PE) from sham (**A**), castration (**B**) and castration + T (**C**) groups, respectively. For typical tracings, the *x*‐axis represents time (min.), while the *y*‐axis represents force (mg). Panel **D** is summary graph of JTE‐013‐induced relaxing effects on rat CCSM *in vitro* from all experimental groups. The stable response to PE stimulation was taken as 100%, while the relaxant effects of JTE‐013 were evaluated as a percentage of this response. *P <* *0.05 *versus* sham or castration + T. (*n* = CC strips obtained from 4 to 8 different rats for each group).

**Figure 9:**
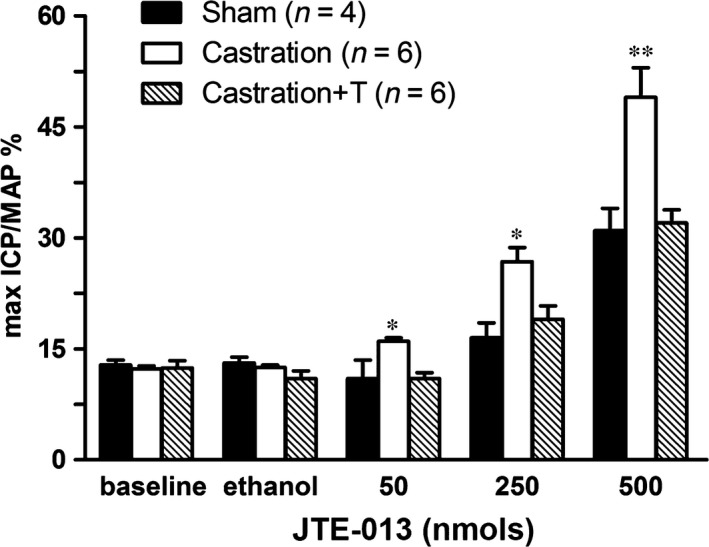
Effect of JTE‐013 (S1P receptor antagonists) on rat corpus cavernosum smooth muscle (CCSM) *in vivo*. A summary bar graph of rat intracavernous pressure (ICP)/mean arterial pressure (MAP) induced by intracavernous injection of accumulative doses (50–500 nmols) of JTE‐013 for all experimental groups (*n* = 4–6 different rats for each group). Baseline ICP and ICP induced by vehicle (50 μl ethanol) are also plotted. Values are expressed as means ± S.E. *P <* *0.05 *versus* sham or castration + T. **P <* *0.01 *versus* sham or castration + T.

In line with our previous study 5, castrated rats were found to exhibit significant ED, with the ICP rise elicited by ES of the cavernous nerve significantly attenuated in the castration group at all stimulation frequencies when compared to controls (Fig. 4). The impaired EF can be partially attributed to increased CCSM tone mediated by vasoconstrictors. In this study, major penile neurotransmitter PE and new ‘vasoactive’ substance S1P were investigated. Consistently, cavernosal strips from castrated animals displayed heighted contractile responses to cumulative doses of PE, which was demonstrated in Figure 5 including typical force tracings (Fig. 5A–C) and the averaged results summarized in the graph of Figure 5D. As shown in Figure 5D, isolated CC strips from castrated rats almost reached fivefold KCl induced contraction at 10^−4^ M PE, whereas sham and T replacement group generated around threefold KCl induced contraction. Similar to our previous report 20, micromolar concentrations of S1P (Fig. 6A and D) and FTY720‐P (Fig. 7A and D) dose‐dependently increased sham rat CC tension with 20 μM S1P and 2 μM FTY720‐P elicited maximal contraction reaching 15% and 20% KCl induced force, respectively. These tensions were enhanced by castration. Maximal contraction induced by 20 μM SIP (Fig. 6B and D) and 2 μM FTY720‐P (Fig. 7B and D) increased by almost twofold and threefold in castrated rats. Again, T administration fully restored both S1P (Fig. 6C and D) and FTY720‐P (Fig. 7C and D) contractility. S1P receptors functional activity and its modulation by androgens were further determined with S1P2 specific inhibitor JTE‐013. As shown in Figure 8, JTE‐013 potently and dose‐dependently relaxed 1 μM PE pre‐contracted CC strips with maximal 75% relaxation. Interestingly, JTE‐013 exhibited more efficacy in relaxing CC strips from castrated rats at the lower concentration of 1 μM but no difference was noted at 5 and 10 μM concentration which attenuated the contraction of both preparations by around 40% and 80%, respectively. Consistent with our *in vitro* observations, JTE‐013 ICI dose‐dependently induced ICP increase, while the solvent (ethanol) did not have any effect on ICP (Fig. 9). Moreover, castration strengthened the JTE‐013 induced erectile response with higher ICP/MAP than normal rats at all doses (50–500 nmols). Additionally, T supplement restored JTE‐013 both *in vitro* and *in vivo* effect.

## Discussion

The current study demonstrated, for the first time, that testosterone modulated the expression and functional activities of S1P receptors in CC. Castration increased serum S1P concentration and up‐regulated the expression of S1P2‐3 receptors while down‐regulated the expression of S1P1 receptor in CC at mRNA level. Accordingly, CCSM contraction induced by agonizing S1P receptors and relaxation mediated by antagonizing S1P receptors were augmented at functional level.

In accordance with our previous studies 5, 31, the surgically castrated rat model was validated by the significant weight loss of the androgen‐sensitive organs (prostate and seminal vesicle), which was recovered after T supplementation. Also in line with previous observations 29, 36, 37, 38, 39, the current study showed T deprivation‐produced ED which was confirmed by the dramatically attenuated ICP/MAP elicited by ES of the cavernous nerve of castrated rats. This hypogonadism‐related ED can be partially attributed to increased CCSM tone mediated by vasoconstrictors. Indeed, our *in vitro* contractility studies observed castration enhanced the α1‐adrenergic agonist PE‐induced rat CCSM contractile responses. Similarly, Wingard *et al*. 30 showed that isolated CC strips from castrated rats were more sensitive to PE stimulation. Although α‐adrenergic signalling pathway is the main contributor responsible for maintaining penis in the flaccid state 40, antagonizing this system with α‐blocker such as yohimbine and phentolamine possessed unsatisfactory efficacy for the treatment of ED 41, 42. Therefore, other vasoconstrictors may play more important roles in hypogonadism ED.

S1P represents one of the key new additions to the list of ‘vasoactive’ substances that modulate vascular tone, as well as many other biological processes including cell proliferation, differentiation, survival and migration 6. In the current study, rat serum S1P level reached over 1 ng/μl and it was increased by castration. It is widely recognized that serum S1P is secreted by activated thrombocytes and erythrocytes 9. Campelo *et al*. 43 found T inhibited platelet activation and aggregation which was likely dependent on endothelial NO synthesis. Li *et al*. 44, 45 also observed platelet aggregation was enhanced in castrated rats when compared with sham and dihydrotestosterone replacement suppressed the increased platelet aggregation in castrated animals, indicating T might modulate S1P level through inhibiting platelet activation. However, it is known that tissue S1P levels are much lower and there is a large concentration gradient maintained between serum and extra vascular compartment 9. Although we did not measure the S1P level in CC due to the difficulty of measuring tissue S1P concentration with HPLC, the critical enzymes for S1P formation in CC were detected. Interestingly, T deprivation down‐regulated SphK1 expression but up‐regulated SphK2. Both SphK1 and SphK2 could alter S1P level. Allende *et al*. 46 and Zemann *et al*. 47 found S1P levels were reduced by 50% in SphK1^−/−^ mice, while Kharel *et al*. 48 showed S1P levels were reduced by only 25% in SphK2^−/−^ mice. Hence, SphK1 played a more important role than SphK2 in maintaining S1P level. Indeed, many studies reported Sphk2 was related to inducing cell apoptosis 49, 50. The reduced SphK1 expression in CC might be negative feedback to up‐regulated serum S1P. The up‐regulation of SphK2 might mainly contribute to the apoptosis in CC which was observed in our previous study 51, rather than the increase in serum S1P concentration. The increased serum S1P level could contribute to hypogonadism ED, although the tissue S1P concentration in CC remains to be elucidated.

Consistent with our previous report 20, the current study found exogenous S1P and its analogue FTY720‐P potently and dose‐dependently induced rat CCSM contraction. Moreover, castration enhanced both S1P and FTY720‐P‐mediated CCSM contractive reaction, which could attribute to increased S1P receptors activity. Indeed, we found T deprivation up‐regulated the expression of S1P2‐3 receptors while down‐regulated the expression of S1P1 receptor in CC at mRNA level. Although S1P1 receptor was thought to mediate vasorelaxation through eNOS pathways 6, 8, 15, S1P1 receptor‐specific agonist SEW2781 was not observed to induce CCSM relaxation and contraction in our study (data not shown). Additionally, we observed S1P2 receptor‐specific antagonist JTE‐013, independent of NO, potently relaxed rat CC pre‐contracted with PE in a dose‐dependent manner. And JTE‐013 exhibited more efficacy in relaxing CC strips from castrated rats at the lower concentration of 1 μM. The contractility of S1P system was further confirmed by our *in vivo* study. In a dose‐dependent manner, ICI of JTE‐013 alone antagonizing S1P2 receptor induced ICP rise. Consistent with our *in vitro* experiment, castrated rats were more sensitive to JTE‐013 with almost 10‐20% higher ICP/MAP observed in a range of 50–500 nmols. Therefore, the augmented expression of S1P2‐3 receptors could mainly contribute to the hyper‐responsiveness to S1P stimulation for castrated animals. Similarly, several other studies demonstrated that S1P system expression and functional activity were modulated by sex hormone. Hemmings *et al*. 52 found that the expression of S1P1 was reduced in mesenteric resistance arteries from aged female rats with lower oestrogen level and restored with oestrogen replacement. And ovariectomy reduced the maximum S1P‐induced vasoconstriction observed in aged rats. Moreover, Guo *et al*. 53 found plasma S1P levels were significantly higher in women than in men, and 17β‐estradiol treatment increased S1P level in EA.hy926 cells. T supplementation recovered all aforementioned alterations including the expression and functional activities of S1P receptors.

Several limitations were noted in the present study. Firstly, S1P itself *in vivo* effect was not determined in this study because the vehicle (0.3 M NaOH) was not suitable for intracaverous injection. Secondly, as specific S1P receptor agonists or antagonists are limited or commercially available and hard to dissolve these lipids, the effect of T on S1P receptors functional activities in CC is not fully evaluated. Additionally, Western blot was not performed because of the low affinity of antibody to S1P receptors. However, our functional studies further confirmed the changed expression of S1P receptors.

In conclusion, we provide novel data that T positively regulate S1P system at both mRNA and functional level. It is suggested that S1P system dysregulation may contribute to hypogonadism‐related ED, and antagonizing S1P receptors in CC might be a new target for treating ED.

## Disclosures

The authors have nothing to disclose.

## Conflict of interest

The authors confirm that there are no conflicts of interest.

## References

[1] Andersson KE , Wagner G . Physiology of penile erection. Physiol Rev. 1995; 75: 191–236. 7831397 10.1152/physrev.1995.75.1.191

[2] Disanto ME . Contractile mechanisms in diabetes‐related erectile dysfunction. Curr Pharm Des. 2005; 11: 3995–4010. 16378506 10.2174/138161205774913417

[3] Zhang XH , Melman A , Disanto ME . Update on corpus cavernosum smooth muscle contractile pathways in erectile function: a role for testosterone? J Sex Med. 2011; 8: 1865–79. 21324096 10.1111/j.1743-6109.2011.02218.x

[4] Zhang XH , Filippi S , Morelli A , *et al* Testosterone restores diabetes‐induced erectile dysfunction and sildenafil responsiveness in two distinct animal models of chemical diabetes. J Sex Med. 2006; 3: 253–66. 16490018 10.1111/j.1743-6109.2006.00207.x

[5] Zhang XH , Morelli A , Luconi M , *et al* Testosterone regulates PDE5 expression and *in vivo* responsiveness to tadalafil in rat corpus cavernosum. Eur Urol. 2005; 47: 409–16. 15716209 10.1016/j.eururo.2004.10.021

[6] Igarashi J , Michel T . Sphingosine‐1‐phosphate and modulation of vascular tone. Cardiovasc Res. 2009; 82: 212–20. 19233865 10.1093/cvr/cvp064 PMC2674011

[7] Liu H , Chakravarty D , Maceyka M , *et al* Sphingosine kinases: a novel family of lipid kinases. Prog Nucleic Acid Res Mol Biol. 2002; 71: 493–511. 12102559 10.1016/s0079-6603(02)71049-0

[8] Schuchardt M , Tolle M , Prufer J , *et al* Pharmacological relevance and potential of sphingosine 1‐phosphate in the vascular system. Br J Pharmacol. 2011; 163: 1140–62. 21309759 10.1111/j.1476-5381.2011.01260.x PMC3144531

[9] Hanel P , Andreani P , Graler MH . Erythrocytes store and release sphingosine 1‐phosphate in blood. Faseb j. 2007; 21: 1202–9. 17215483 10.1096/fj.06-7433com

[10] Hla T . Physiological and pathological actions of sphingosine 1‐phosphate. Semin Cell Dev Biol. 2004; 15: 513–20. 15271296 10.1016/j.semcdb.2004.05.002

[11] Hla T , Lee MJ , Ancellin N , *et al* Lysophospholipids–receptor revelations. Science. 2001; 294: 1875–8. 11729304 10.1126/science.1065323

[12] Graler MH , Bernhardt G , Lipp M . EDG6, a novel G‐protein‐coupled receptor related to receptors for bioactive lysophospholipids, is specifically expressed in lymphoid tissue. Genomics. 1998; 53: 164–9. 9790765 10.1006/geno.1998.5491

[13] Im DS , Heise CE , Ancellin N , *et al* Characterization of a novel sphingosine 1‐phosphate receptor, Edg‐8. J Biol Chem. 2000; 275: 14281–6. 10799507 10.1074/jbc.275.19.14281

[14] Michel MC , Mulders AC , Jongsma M , *et al* Vascular effects of sphingolipids. Acta Paediatr. 2007; 96: 44–8. 10.1111/j.1651-2227.2007.00207.x 17391441

[15] Igarashi J , Bernier SG , Michel T . Sphingosine 1‐phosphate and activation of endothelial nitric‐oxide synthase. differential regulation of Akt and MAP kinase pathways by EDG and bradykinin receptors in vascular endothelial cells. J Biol Chem. 2001; 276: 12420–6. 11278407 10.1074/jbc.M008375200

[16] Aydin M , Downing K , Villegas G , *et al* The sphingosine‐1‐phosphate pathway is upregulated in response to partial urethral obstruction in male rats and activates RhoA/Rho‐kinase signalling. BJU Int. 2010; 106: 562–71. 20128782 10.1111/j.1464-410X.2009.09156.x

[17] Sandhu KS , Chua RG , Zhang X , *et al* Regional heterogeneity in expression of the sphingosine‐1‐phosphate pathway in the female rat lower urinary tract. Am J Obstet Gynecol. 2009; 200: 576: E1‐7. 19254791 10.1016/j.ajog.2008.12.007

[18] Chiba Y , Suzuki K , Uechi M , *et al* Downregulation of sphingosine‐1‐phosphate receptors in bronchial smooth muscle of mouse experimental asthma. Pharmacol Res. 2010; 62: 357–63. 20554039 10.1016/j.phrs.2010.05.005

[19] di Villa Bianca R , Sorrentino R , Sorrentino R , *et al* Sphingosine 1‐phosphate induces endothelial nitric‐oxide synthase activation through phosphorylation in human corpus cavernosum. J Pharmacol Exp Ther. 2006; 316: 703–8. 16234413 10.1124/jpet.105.093419

[20] Zhang XH , Kuppam D , Melman A , *et al* The sphingosine‐1‐phosphate synthetic analogue fingolimod (FTY720‐P) potently modulates rat and human corpus cavernosum smooth muscle tone *via* the S1P3 receptor both *in vitro* and *in vivo* . J Sex Med. 2010; 7: 7–8.

[21] Maggi M , Filippi S , Ledda F , *et al* Erectile dysfunction: from biochemical pharmacology to advances in medical therapy. Eur J Endocrinol. 2000; 143: 143–54. 10913932 10.1530/eje.0.1430143

[22] Morelli A , Filippi S , Mancina R , *et al* Androgens regulate phosphodiesterase type 5 expression and functional activity in corpora cavernosa. Endocrinology. 2004; 145: 2253–63. 14764637 10.1210/en.2003-1699

[23] Andersson KE . Erectile physiological and pathophysiological pathways involved in erectile dysfunction. J Urol. 2003; 170: S6–14. 10.1097/01.ju.0000075362.08363.a4 12853766

[24] Bivalacqua TJ , Usta MF , Champion HC , *et al* Endothelial dysfunction in erectile dysfunction: role of the endothelium in erectile physiology and disease. J Androl. 2003; 24: S17–37. 14581492 10.1002/j.1939-4640.2003.tb02743.x

[25] Lin CS , Lin G , Lue TF . Cyclic nucleotide signaling in cavernous smooth muscle. J Sex Med. 2005; 2: 478–91. 16422842 10.1111/j.1743-6109.2005.00080.x

[26] Filippi S , Morelli A , Sandner P , *et al* Characterization and functional role of androgen‐dependent PDE5 activity in the bladder. Endocrinology. 2007; 148: 1019–29. 17138653 10.1210/en.2006-1079

[27] Morelli A , Filippi S , Zhang XH , *et al* Peripheral regulatory mechanisms in erection. Int J Androl. 2005; 28(Suppl 2): 23–7. 16236060 10.1111/j.1365-2605.2005.00550.x

[28] Traish AM , Park K , Dhir V , *et al* Effects of castration and androgen replacement on erectile function in a rabbit model. Endocrinology. 1999; 140: 1861–8. 10098525 10.1210/endo.140.4.6655

[29] Reilly CM , Stopper VS , Mills TM . Androgens modulate the alpha‐adrenergic responsiveness of vascular smooth muscle in the corpus cavernosum. J Androl. 1997; 18: 26–31. 9089065

[30] Wingard CJ , Johnson JA , Holmes A , *et al* Improved erectile function after Rho‐kinase inhibition in a rat castrate model of erectile dysfunction. Am J Physiol Regul Integr Comp Physiol. 2003; 284: R1572–9. 12573976 10.1152/ajpregu.00041.2003

[31] Zhang X , Zang N , Wei Y , *et al* Testosterone regulates smooth muscle contractile pathways in the rat prostate: emphasis on PDE5 signaling. Am J Physiol Endocrinol Metab. 2012; 302: E243–53. 22028410 10.1152/ajpendo.00458.2011 PMC3340899

[32] Chua RG , Calenda G , Zhang X , *et al* Testosterone regulates erectile function and Vcsa1 expression in the corpora of rats. Mol Cell Endocrinol. 2009; 303: 67–73. 19428993 10.1016/j.mce.2009.02.001 PMC2694216

[33] Livak KJ , Schmittgen TD . Analysis of relative gene expression data using real‐time quantitative PCR and the 2(‐Delta Delta C(T)) Method. Methods. 2001; 25: 402–8. 11846609 10.1006/meth.2001.1262

[34] Zhang X , Kuppam DS , Melman A , *et al* *In vitro* and *in vivo* relaxation of urinary bladder smooth muscle by the selective myosin II inhibitor, blebbistatin. BJU Int. 2011; 107: 310–7. 20482706 10.1111/j.1464-410X.2010.09366.x

[35] Melman A , Biggs G , Davies K , *et al* Gene transfer with a vector expressing Maxi‐K from a smooth muscle‐specific promoter restores erectile function in the aging rat. Gene Ther. 2008; 15: 364–70. 18200069 10.1038/sj.gt.3303093

[36] Palese MA , Crone JK , Burnett AL . A castrated mouse model of erectile dysfunction. J Androl. 2003; 24: 699–703. 12954660 10.1002/j.1939-4640.2003.tb02729.x

[37] Baba K , Yajima M , Carrier S , *et al* Delayed testosterone replacement restores nitric oxide synthase‐containing nerve fibres and the erectile response in rat penis. BJU Int. 2000; 85: 953–8. 10792181 10.1046/j.1464-410x.2000.00598.x

[38] Mills TM , Stopper VS , Wiedmeier VT . Effects of castration and androgen replacement on the hemodynamics of penile erection in the rat. Biol Reprod. 1994; 51: 234–8. 7948478 10.1095/biolreprod51.2.234

[39] Traish AM , Munarriz R , O'Connell L , *et al* Effects of medical or surgical castration on erectile function in an animal model. J Androl. 2003; 24: 381–7. 12721214 10.1002/j.1939-4640.2003.tb02686.x

[40] DiSanto ME . Corpus cavernosum smooth muscle physiology: a role for sex hormones? J Androl. 2003; 24: S6–16. 14581491 10.1002/j.1939-4640.2003.tb02742.x

[41] Andersson KE , Stief C . Oral alpha adrenoceptor blockade as a treatment of erectile dysfunction. World J Urol. 2001; 19: 9–13. 11289574 10.1007/pl00007093

[42] Morales A . Yohimbine in erectile dysfunction: would an orphan drug ever be properly assessed? World J Urol. 2001; 19: 251–5. 11550783 10.1007/s003450000182

[43] Campelo AE , Cutini PH , Massheimer VL . Testosterone modulates platelet aggregation and endothelial cell growth through nitric oxide pathway. J Endocrinol. 2012; 213: 77–87. 22281525 10.1530/JOE-11-0441

[44] Li S , Li X , Li J , *et al* Experimental arterial thrombosis regulated by androgen and its receptor *via* modulation of platelet activation. Thromb Res. 2007; 121: 127–34. 17451792 10.1016/j.thromres.2007.03.008

[45] Li S , Li X , Li J , *et al* Inhibition of oxidative‐stress‐induced platelet aggregation by androgen at physiological levels *via* its receptor is associated with the reduction of thromboxane A2 release from platelets. Steroids. 2007; 72: 875–80. 17825336 10.1016/j.steroids.2007.07.007

[46] Allende ML , Sasaki T , Kawai H , *et al* Mice deficient in sphingosine kinase 1 are rendered lymphopenic by FTY720. J Biol Chem. 2004; 279: 52487–92. 15459201 10.1074/jbc.M406512200

[47] Zemann B , Kinzel B , Muller M , *et al* Sphingosine kinase type 2 is essential for lymphopenia induced by the immunomodulatory drug FTY720. Blood. 2006; 107: 1454–8. 16223773 10.1182/blood-2005-07-2628

[48] Kharel Y , Lee S , Snyder AH , *et al* Sphingosine kinase 2 is required for modulation of lymphocyte traffic by FTY720. J Biol Chem. 2005; 280: 36865–72. 16093248 10.1074/jbc.M506293200

[49] Kamada K , Arita N , Tsubaki T , *et al* Expression of sphingosine kinase 2 in synovial fibroblasts of rheumatoid arthritis contributing to apoptosis by a sphingosine analogue, FTY720. Pathol Int. 2009; 59: 382–9. 19490468 10.1111/j.1440-1827.2009.02381.x

[50] Abdel Hadi L , Di Vito C , Marfia G , *et al* Sphingosine kinase 2 and ceramide transport as key targets of the natural flavonoid luteolin to induce apoptosis in colon cancer cells. PLoS ONE. 2015; 10: e0143384. 26580959 10.1371/journal.pone.0143384 PMC4651545

[51] Zhang XH , Hu LQ , Zheng XM , *et al* Apoptosis in rat erectile tissue induced by castration. Asian J Androl. 1999; 1: 181–5. 11225891

[52] Hemmings DG , Xu Y , Davidge ST . Sphingosine 1‐phosphate‐induced vasoconstriction is elevated in mesenteric resistance arteries from aged female rats. Br J Pharmacol. 2004; 143: 276–84. 15326035 10.1038/sj.bjp.0705752 PMC1575332

[53] Guo S , Yu Y , Zhang N , *et al* Higher level of plasma bioactive molecule sphingosine 1‐phosphate in women is associated with estrogen. Biochim Biophys Acta. 2014; 1841: 836–46. 24603322 10.1016/j.bbalip.2014.02.005

